# Supplementary material for: Novel Spike-stabilized trimers with improved production protect K18-hACE2 mice and golden Syrian hamsters from the highly pathogenic SARS-CoV-2 Beta variant
Source: Front Immunol. 2023 Dec 4;14:1291972. doi: 10.3389/fimmu.2023.1291972 (PMC10731958; doi:10.3389/fimmu.2023.1291972)
Supplement: Supplementary file 1 [file DataSheet_1.pdf]

frontiers

# *Supplementary Material*

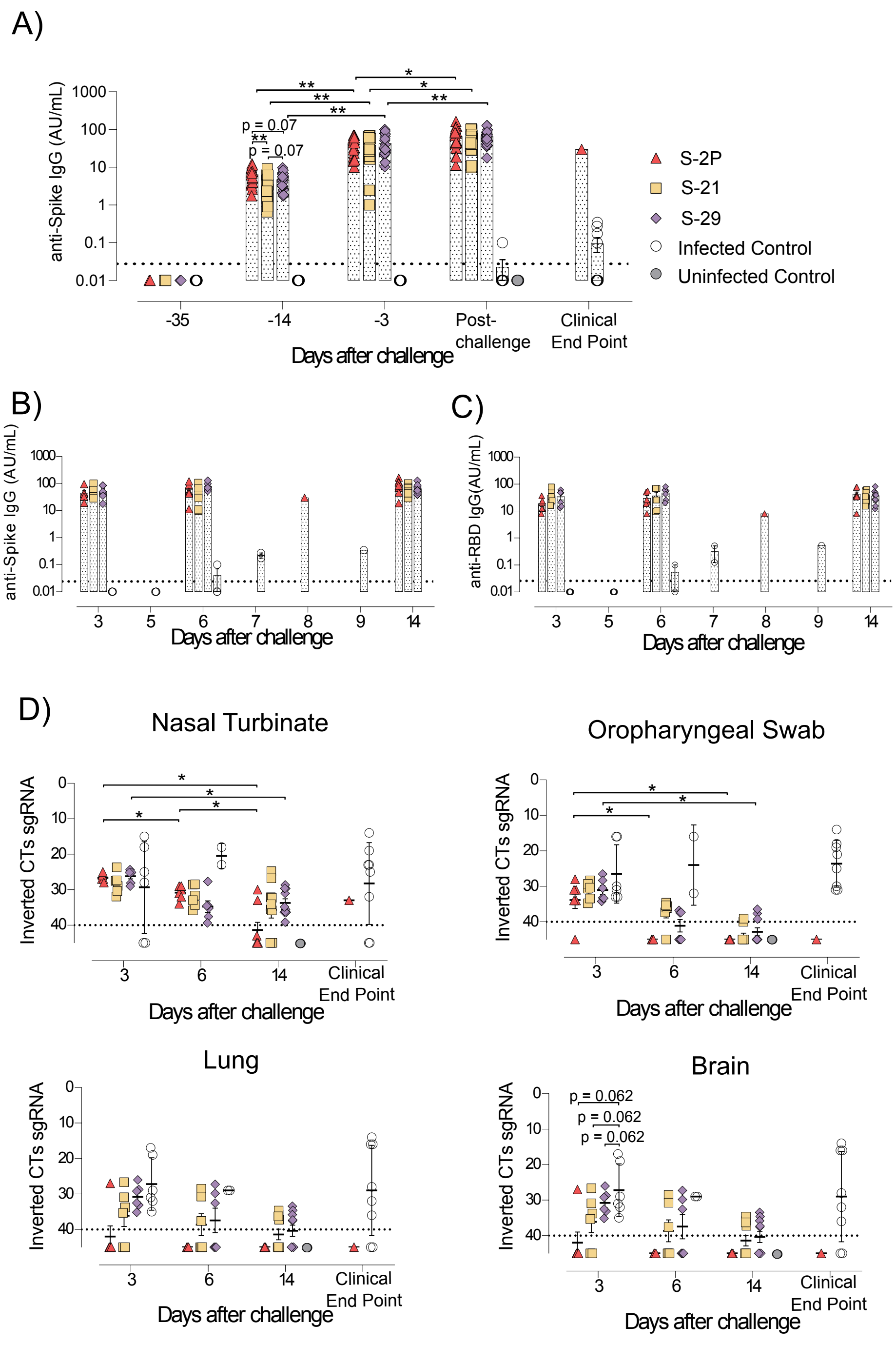


**Supplementary Figure 1. Vaccine-induced anti-S IgG responses and levels of tissue sub-genomic RNA in immunized K18-hACE2 transgenic mice after challenge with SARS-CoV-2 B.1.351 (Beta) variant.**

**A** Kinetics of anti-S IgG antibodies in serum samples. Red triangles: S-2P group (n=21). Yellow squares: S-21 (n=22). Purple diamond: S-29 (n=22). White circles: unvaccinated-challenge mice (n=16). Grey circles: unvaccinated-uninfected mice (n=10). Groups in each time point were analyzed

using Kruskal-Wallis and Conover's post-hoc tests with multiple comparison correction by FDR. Differences among animals within a particular group along time were analyzed using the Friedman test corrected for multiple comparison using FDR. **B** Kinetics of anti-S and anti-RBD IgG antibodies in serum samples after SARS-CoV-2 B.1.351 challenge. Mean plus standard error of the mean (SEM) is shown. **C** Levels of SARS-CoV-2 subgenomic RNA (represented as inverted Ct) in oropharyngeal swabs, nasal turbinate, lung, and brain after virus challenge. Dotted line indicates limit of detection (40 Cts). Differences among groups were analyzed using Peto & Peto left-censored k-sample test corrected by FDR. * $p<0.05$, ** $p<0.01$.

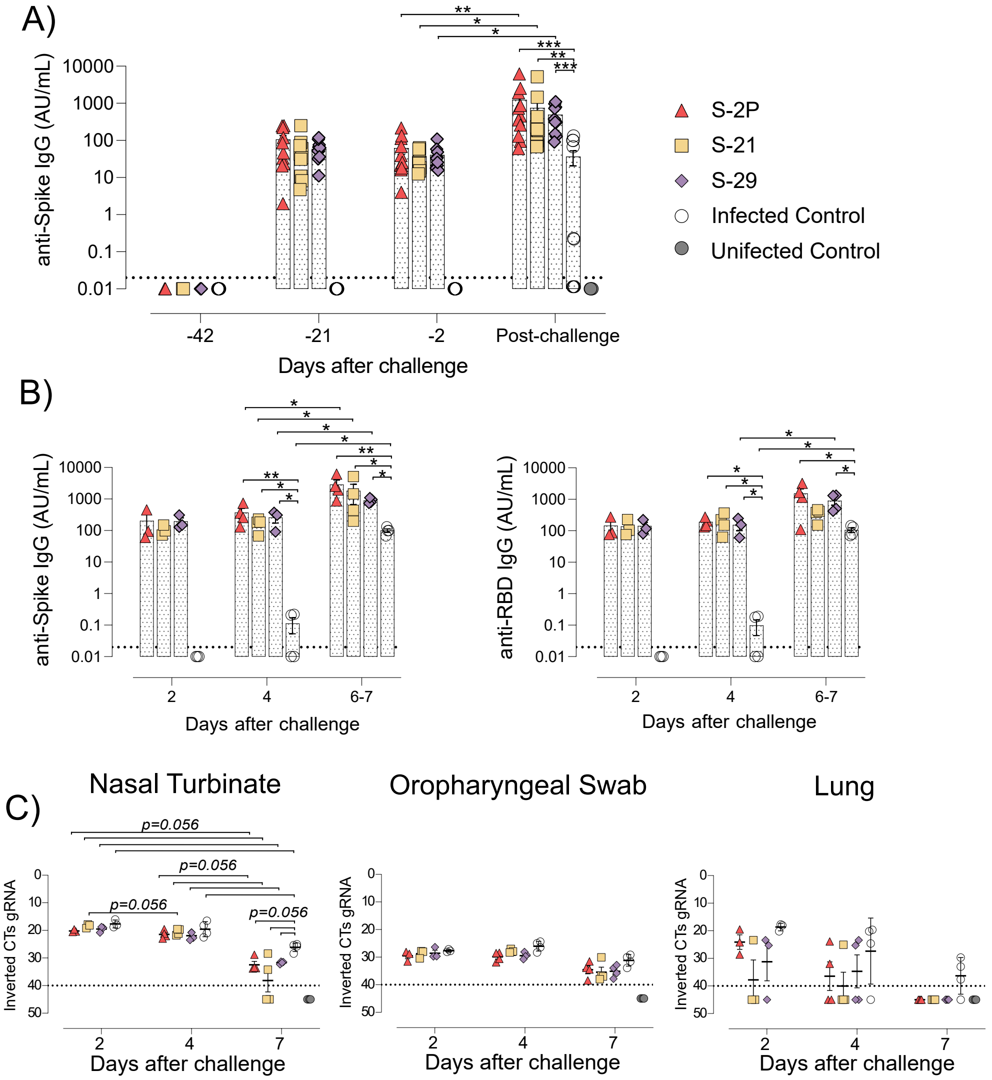


**Supplementary Figure 2. Anti-S IgG responses and levels of sub-genomic RNA in tissues of immunized GSH after challenge with SARS-CoV-2 B.1.351 (Beta) variant.**

**A** Kinetics of anti-S antibodies in serum samples. Red triangles: S-2P group (n= 11). Yellow squares: S-21 (n=11). Purple diamond: S-29 (n=11). White circles: unvaccinated-challenge mice (n=11). Grey circles: unvaccinated-uninfected mice (n=5). Groups in each time point were analyzed using Kruskal-Wallis and Conover's post-hoc test with multiple comparison correction by FDR. Differences among animals within a particular group along time were analyzed using the Friedman test corrected for multiple comparison using FDR. **B** Kinetics of anti-S and anti-RBD IgG antibodies in serum samples at days 2, 4 and 6-7 (end point) after SARS-CoV-2 Beta challenge. Mean plus standard error of the

means (SEM) are shown. **C** Levels of SARS-CoV-2 subgenomic RNA (represented as inverted Ct) in oropharyngeal swabs, nasal turbinate, and lung after virus challenge. Dotted line indicates limit of positivity (40 Cts). Differences among groups were analyzed using Peto & Peto Left-censored k-sample test corrected by FDR. * $p<0.05$, ** $p<0.01$, *** $p<0.001$.
